# Therapeutic potential of fucoidan in the reduction of hepatic pathology in murine schistosomiasis japonica

**DOI:** 10.1186/s13071-020-04332-7

**Published:** 2020-09-07

**Authors:** Xueqi Bai, Maining Li, Xinyue Wang, Hao Chang, Yangyue Ni, Chen Li, Kaiyue He, Huiquan Wang, Yuxuan Yang, Tian Tian, Min Hou, Minjun Ji, Zhipeng Xu

**Affiliations:** 1 Department of Pathogen Biology, Jiangsu Province Key Laboratory of Modern Pathogen Biology, Nanjing Medical University, Nanjing, Jiangsu 211166 China; 2 Department of Dermatology, The Affiliated Sir Run Run Hospital of Nanjing Medical University, Nanjing, Jiangsu 211100 China

**Keywords:** Granuloma, Fibrosis, Fucoidan, Treg, *Schistosoma japonicum*

## Abstract

**Background:**

Hepatic granuloma formation and fibrosis as the consequence of tissue entrapped eggs produced by female schistosomes characterize the pathology of *Schistosoma japonicum* infection. It has been proposed that fucoidan, a sulfated polysaccharide existing naturally in brown seaweed *Fucus vesiculosus*, plays a diversified role to perform immunomodulatory activities. However, whether fucoidan functions in the host hepatic pathology is unknown and identifying the potential mechanism that is responsible for hepatic improvement is still necessary.

**Methods:**

We evaluated the hepatic pathology from *S. japonicum*-infected mice after treatment with fucoidan. qRT-PCR and immunofluorescence were used to detect the pro- or anti-inflammatory factors and the phosphorylated p65 in the livers. In addition, flow cytometry was also performed to investigate the T cell subsets in the *S. japonicum*-infected mice after treatment with fucoidan, and functional molecules relatively specific to Treg cells were detected *in vitro*. Furthermore, macrophages were treated with fucoidan *in vitro* and to detect the inflammatory cytokines.

**Results:**

Treatment with fucoidan significantly reduced the hepatic granuloma size and fibrosis response during *S. japonicum* infection. The attenuated phospho-p65 protein levels and the mRNA levels of pro-inflammatory cytokines (IL-6, IL-12 and TNF-α) were observed in the livers from fucoidan-treated *S. japonicum-*infected mice; however, the mRNA levels of anti-inflammatory cytokines (IL-4 and IL-13) were increased. In addition, the infiltration of Treg cells was significantly enhanced both in the livers and spleens from fucoidan-treated *S. japonicum-*infected mice. Consistent with this, the mRNA levels of IL-10 and TGF-β were dramatically increased in the livers from *S. japonicum-*infected mice after fucoidan treatment. Furthermore, *in vitro* stimulated splenocytes with fucoidan resulted in increasing Treg cells in splenocytes as well as the functional expression of CC chemokine receptor type 4 (CCR4) and CXC chemokine receptor type 5 (CXCR5) in Treg cells. Additionally, fucoidan promoted the mRNA levels of IL-4 and IL-13 in macrophages.

**Conclusions:**

These findings suggest an important role of natural fucoidan in reducing hepatic pathology in the progress of *S. japonicum* infection with a stronger Treg response, which may reveal a new potential therapeutic strategy for hepatic disease caused by parasitic chronic infection.
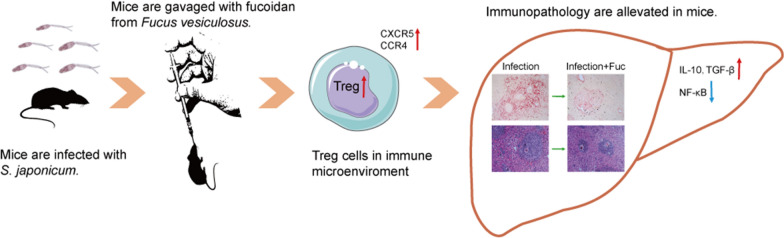

## Background

Schistosomiasis, caused by the parasite of the genus *Schistosoma*, is a chronic helminth disease remaining in tropical and subtropical developing countries [1]. The main pathological manifestations of schistosomiasis japonica are granuloma and subsequent fibrosis due to lodged worm eggs, resulting in irreversible, lethal impairment in the host liver and intestine [2, 3]. Numerous studies suggested that the subsets of CD4^+^ T cells play critical roles in regulating hepatic immune pathology during *Schistosoma japonicum* infection. A type-2 helper T (Th2) immune response is initially evoked at a low level to keep Th1 inflammatory pathology in check during the acute stage [4], however, it turns out an interleukin-4 (IL-4)/IL-13-driven granulomatous response chronically [2]. Th17 cells also function during the late infection stage together with Th2 cells [2]. Notably, CD4^+^CD25^+^Foxp3^+^ regulatory T (Treg) cells have been demonstrated to diminish excessive Th2 anti-egg response in a transforming growth factor-β (TGF-β)- and-IL-10-dependent manner, thus reducing schistosome egg-induced hepatic immune pathology [5, 6].

Macrophages act as a key cell population during the development of liver fibrosis in schistosomiasis japonica. Studies have identified that macrophages were recruited specifically to the developing egg-induced granuloma in the liver and account for a prominent constituent as much as 30% [7]. Importantly, consistent with egg deposition promoting Th2 responses, macrophages are observed to be an alternatively activated phenotype (M2) rather than a classically activated one (M1) with observed upregulation of Arg-1, Ym-1, Fizz-1 and downregulation of IFN-γ during the chronic phase of the infection [2, 8, 9]. In addition, M2 macrophages play crucial roles in the immune response in that they can contribute to Th2-type responses and are essential for the downmodulation of Th1 responses [10, 11]. More thought-provoking evidence is that macrophages express high levels of TGF-β and IL-10, simultaneously increase the proportion of CD4^+^CD25^+^Foxp3^+^ Treg cells during *S. japonicum* infection [5].

Fucoidan, which is extracted mainly from brown marine algae, is a polysaccharide containing substantial L-fucose and sulfate ester groups [12]. It is well established that fucoidan has been reported to show various biological activities both in *vivo* and *vitro* such as anti-lipid accumulation and anti-tumor [13, 14], as well as anti-inflammatory immune responses [15]. Recent study has shown that fucoidan inhibited LPS-induced inflammation in macrophages by blocking the toll-like receptor-4 (TLR4) nuclear factor-κB (NF-κB) signal pathway [16]. Fucoidan significantly reduced expressions of cytokines (TIMP-1, CXCL1, MCP-1 and MIP-2), consequently attenuated pneumonia and lung fibrosis in a mice model [17]. However, whether fucoidan could impact on the inflammation-associated hepatic pathologies remains unclear.

In this study, we show that the natural biological product-derived fucoidan exerts anti-hepatic pathology and anti-inflammatory activity, which is associated with the inducing hepatic Treg cells. Our study suggests a new potential therapeutic strategy at the acute phase of schistosomiasis may result in a mild course of murine schistosomiasis and can be a promising complementary treatment, reverting sequelae of such infection.

## Methods

### Animals and parasites preparation

Six-week-old female C57BL/6 mice obtained from the Animal Core Facility of Nanjing Medical University were bred in a specific pathogen-free (SPF) environment.

*Oncomelania hupensis* snails infected with *S. japonicum* from which cercariae were collected, were the kind gifts provided by the Jiangsu Institute of Parasitic Diseases (Wuxi, Jiangsu, China).

### *Schistosoma japonicum* infection and experimental animal model

Twenty mice were weighed and randomly divided into four groups (*n* = 5 for each group) labeled with Ctrl (normal mice without any treatment), Ctrl + Fuco (normal mice with fucoidan treatment), Infection (*S. japonicum* infection without any treatment), and Infection + Fuco (*S. japonicum* infection with fucoidan treatment), respectively.

**Fig. 1:**
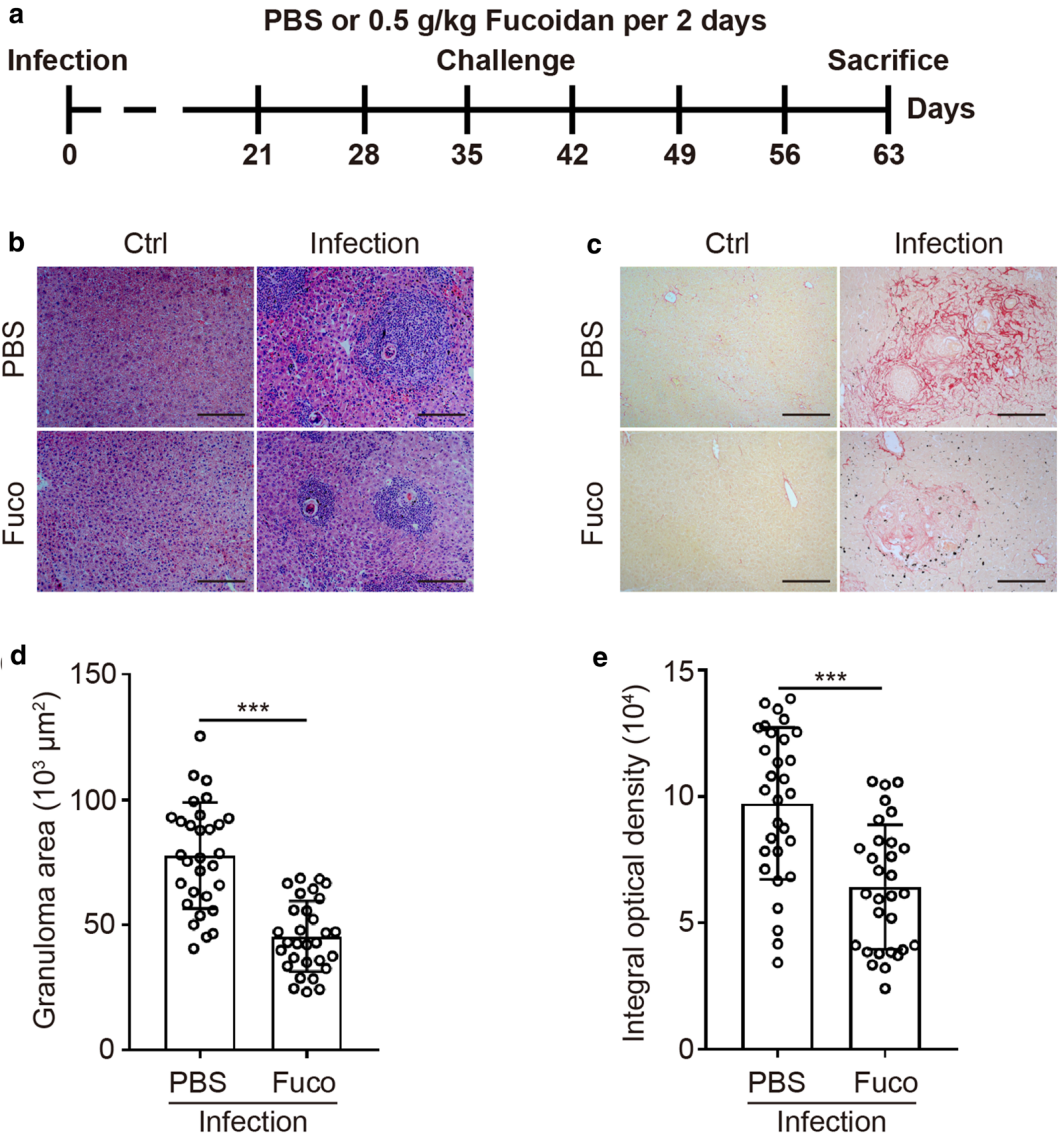
Hepatic pathology was detected after fucoidan treatment during *S. japonicum* infection. **a** Experimental procedure of *S. japonicum*-infected model. Initially infected with *S. japonicum* cercariae percutaneously on day 0, mice were treated with fucoidan or PBS by intragastric administration, 500 mg/kg per 2 days since day 21. Mice were sacrificed at 8 weeks for further study. **b**, **d** Paraffin-embedded liver sections were stained with hematoxylin and eosin (H&E). Original magnification: ×100. For each group, the sizes of 30 liver granulomas around single eggs (six random granulomas in each mouse) were qualified with AxioVision Rel 4.7. **c**, **e** Paraffin-embedded sections were stained with Sirius Red. Original magnification: ×100. The mean optical density of collagen fibers by Sirius Red was digitized and analyzed by Image-Pro-Plus 6.0 software. Data are expressed as the mean ± SD for each group (*n* = 5 for each group), and all experiments were performed twice with similar results. *P < 0.05, **P < 0.01, ***P < 0.001 (Student’s t-test). *Scale-bars*: **b**, **c**, 20 μm

Mice in the Infection and Infection + Fuco groups were infected percutaneously with 12 *S. japonicum* cercariae on day 0. Since the third-week post-infection (day 21), mice in the Ctrl + Fuco group and Infection + Fuco group were treated with fucoidan (from *Fucus vesiculosus*, Sigma-Aldrich, St Louis, MO, USA) dissolved in phosphate-buffered saline (PBS) *via* intragastric administration (500 mg/kg per 2 days) as suggested in a previous study [17]. Meanwhile, mice in the Ctrl group and Infection group were given the equivalent volume of sterile PBS for 6 weeks, following the same schedule that was used for the other groups. Mice were euthanized *via* diethyl ether-induced anesthesia at the 9th-week post-infection (Fig. 1a).

### Liver pathology examination

The livers from each group was perfused with perfusion buffer (0.85% (w/v) NaCl plus 1% (w/v) trisodium citrate) and the hepatic tissue derived from right lobes were collected and fixed in 4% neutralized formaldehyde and embedded in paraffin. Liver sections (5 μm) were dewaxed and stained with hematoxylin and eosin (H&E) for granulomas analysis or Sirius Red (Sigma-Aldrich) for fibrosis analysis.

For each group, 30 random single-egg granulomas around the single egg (6 granulomas each mouse) were calculated using AxioVision Rel 4.7 (Carl Zeiss, Hallberg moss, Germany). Granuloma sizes (×100) are expressed as means of areas measured in mm^2^ ± SD.

Thirty random digital images (×100) were captured from collagen-specific Sirius red-stained slides of each group. The degree of fibrosis was evaluated histologically by measuring the intensity of fibrosis using Image-Pro Plus v6.0 (Media Cybernetics, Silver Spring, MD, USA). Integral optical density by the image area was divided to determine the mean optical density of collagen.

### Immunofluorescence

Immunocytochemical staining was used to detect phospho-NF-κB p65 expression. Frozen liver sections were fixed with 4% ice-cold paraformaldehyde and washed three times in 1× PBS. Tissues were permeabilized with 0.5% Triton X-100 at room temperature for 30 min. 2% Bovine Serum Albumin (Sigma-Aldrich) was used to block unspecific binding sites for 1 h. Then tissues were incubated with antibodies against phospho-p65 (Cell Signaling Technology, Danvers, MA, USA) at 1:200 dilution at 4 °C overnight. Following three washes with PBS, the samples were incubated with PE-conjugated goat anti-rabbit IgG antibody (Cell Signaling Technology) at a 1:200 dilution at room temperature for 2 h. Subsequently, tissues were counterstained with DAPI (Abcam, Cambridge, MA, USA) for nucleus staining.

To detect the expression of CD4^+^Foxp3^+^ Treg cells, frozen liver sections were fixed and permeabilized following the procedure mentioned above. After 1% Bovine Serum Albumin (Sigma-Aldrich) was used to block unspecific binding sites for 1 h, the liver tissues were incubated with antibodies against CD4-FITC (eBioscience, San Diego, CA, USA; at 1:20 dilution) and Foxp3-PE (eBioscience; at 1:20 dilution) for 2 h. Subsequently, tissues were washed and counterstained with DAPI (Abcam) for nucleus staining.

Images were acquired with AxioVision Rel 4.7 (Carl Zeiss). Quantification of fluorescence was achieved by Image Pro Plus software v6.0 (Media Cybernetics).

### Lymphocyte isolation

To prepare splenocytes, spleens were ground in incomplete RPMI 1640 medium (Gibco, Grand Island, NY, USA). Red blood cells (RBCs) were lysed with an RBC lysis solution (Sigma Aldrich). After being washed in staining buffer containing PBS with 1% fetal bovine serum (FBS), cells were filtered through 200-gauge mesh and collected.

Hepatic lymphocytes were prepared as described previously with some modifications [18]. The remaining hepatic tissues were washed in D-Hank’s solution (Invitrogen, Carlsbad, CA, USA) *via* the portal vein, cut into pieces and digested in 20 mg Collagenase B (Invitrogen) at 37 °C for 45 min. The digested tissues were then minced in a homogenized washing buffer containing 1× PBS with 1% FBS and collected. Hepatocytes were sedimented by centrifugation at 300×*g*. The remaining cells were separated into 4 layers by centrifugation at 500×*g* on a 35% Percoll (Sigma-Aldrich) gradient. Lymphocytes on the second layer were subsequently centrifuged at 250×*g*, resuspended in RBC lysis buffer and washed by complete RPMI 1640 medium. Finally, cells were passed through Pre-Separation Filters (20 μm; Miltenyi Biotec, Bergisch Gladbach, Germany) and were collected for further study.

### Cell culture and *in vitro* treatment

Splenocytes were purified from 6-week-old normal C57BL/6 mice as previously described [10]. They were cultured in 6-well plates (2 × 10^6^ per well) in RPMI 1640 medium supplemented with 10% FBS and 1% penicillin/streptomycin (10,000 U/ml penicillin and 10 mg/m streptomycin).

RAW264.7 cells were purchased from the Cell Bank of Chinese Academy of Sciences. Cells were spread into 6-well plates equally (2 × 10^6^ per well), and cultured in DMEM (Gibco) with 10% FBS and 1% penicillin/streptomycin (10,000 U/ml penicillin and 10 mg/ml streptomycin).

Cells were incubated with or without fucoidan (30 μg/ml) at 37 °C with 5% CO_2_ for 24 h [19]. Then, cells were collected for further analysis.

### RNA extraction and quantitative RT-PCR (qRT-PCR)

Total RNA of tissue or cells was extracted using TRIzol Reagent (Invitrogen), and measured using a NanoDrop 2000 spectrophotometer (Thermo Fisher Scientific, Waltham, MA, USA). Complementary DNA was synthesized using HiScript Q RT SuperMix (Vazyme, Nanjing, China). Quantitative real-time polymerase chain reaction (qRT-PCR) was performed using Power SYBR Green PCR Master Mix (Applied Biosystems, Foster City, CA, USA) with the Applied Biosystems^TM^ QuantStudio^TM^ 5 system. The cycling parameters were as follows: hold stage, 95 °C for 3 min; PCR stage, 40 cycles of 95 °C for 10 s and 60 °C for 30 s; melt curve stage, 95 °C for 15 s and 60 °C for 1 min, which was concluded by the melting curve analysis process. Triplicate PCR reactions were performed for each sample. The murine housekeeping gene GAPDH was used to normalize the gene expression using a comparative method (2^−ΔΔCq^) [10]. The primers were designed by Primer Premier 5 software (Premier Biosoft, San Francisco, CA, USA). All primer sequences are listed in Additional file 1: Table S1.

### Flow cytometry

Flow cytometry was conducted as previously described with some modifications [10]. The following antibodies were used for flow cytometry: CD3e-PerCP-Cyanine5.5; CD4-FITC; CD25-APC; IFN-γ-PE; IL-4-PE; IL-17A-PE; Foxp3-PE; PD-1-PE; KLRG1-PE (all from eBioscience, San Diego, CA, USA); CXCR5-PerCP-Cyanine5.5 (BD Pharmingen, San Jose, CA, USA); ICOS-BV421 (BD Pharmingen); and CCR4-PE (BioLegend, San Diego, CA, USA).

To identify Th cell subsets, 2 × 10^6^ cells/ml were cultured in RPMI 1640 containing 10% FBS, and activated by leukocyte activation cocktail with GolgiPlug (BD Pharmingen) for 6 h at 37 °C in 5 % CO_2_. After the incubation, cells were harvested and labeled with surface molecules CD3e-PerCP-Cyanine5.5 (1:200) and CD4-FITC (1:200). After that, cells were washed, fixed and permeabilized by cytofix/cytoperm plus fixation/permeabilization kit (BD Pharmingen), and intracellularly stained with antibodies against IFN-γ-PE, IL-4-PE, or IL-17A-PE (1:50), respectively.

To identify Treg cells, 2 × 10^6^ cells were labeled with CD4-FITC (1:200), CD25-APC (1:100) for 30 min, and then washed, fixed, and permeabilized with fixation-permeabilization buffer (eBioscience). Subsequently blocked by Fc receptor binding inhibitor (eBioscience), cells were intracellularly stained with the antibody against Foxp3-PE (1:50).

To identify suppression function on Treg cells, cells were harvested after 24 h *in vitro* treatment with fucoidan and labeled with CD4-FITC, CD25-APC for 30 min. Meanwhile, cells were stained with antibodies against surface markers, including CCR4-PE, CXCR5-PerCP-Cyanine5.5, PD-1-PE, ICOS-BV421, or KLRG1-PE (1:100), respectively.

Flow cytometry was conducted using a BD FACSVerse^TM^ cytometer (BD Pharmingen) followed by data analysis by FlowJo software (TreeStar Inc., Ashland, OR, USA).

### Statistical analysis

Data obtained from this study are expressed as the mean ± standard deviation (SD). GraphPad Prism v8.0 (GraphPad Software, San Diego, California, USA) was used in statistical analysis. Significant differences between the two experimental groups were identified by unpaired Student’s t-test. Multiple comparisons were performed by one-way analysis of variance (ANOVA) and the least-significant difference (LSD) between every two groups. A value of *P *< 0.05 was considered statistically significant. Significant differences are indicated as follows: *P < 0.05; **P < 0.005; ***P < 0.001.

## Results

### Fucoidan alleviates hepatic pathology during *S. japonicum* infection

Since manifestations such as anti-egg granulomatous responses and fibrosis deposition are typical during *S. japonicum* infection [1], HE and Sirius Red staining were used to investigate the impact of fucoidan on these hepatic pathologies (Fig. 1a). The staining results demonstrated that the mean area of isolated granuloma was significantly declined in *S. japonicum*-infected mice with fucoidan treatment (t-test; *t*_(58)_ = 6.948, *P *< 0.0001) (Fig. 1b, d). In addition, the decrease in granuloma size was accompanied by a decrease in fibrosis in fucoidan-treated mice, as shown by Sirius Red stained sections (t-test; *t*_(58)_ = 4.652, *P *< 0.0001) (Fig. 1c, e). These results suggest that fucoidan played a critical role in moderating hepatic pathology during *S. japonicum* infection.

### Fucoidan downregulates hepatic inflammation in *S. japonicum*-infected mice

**Fig. 2:**
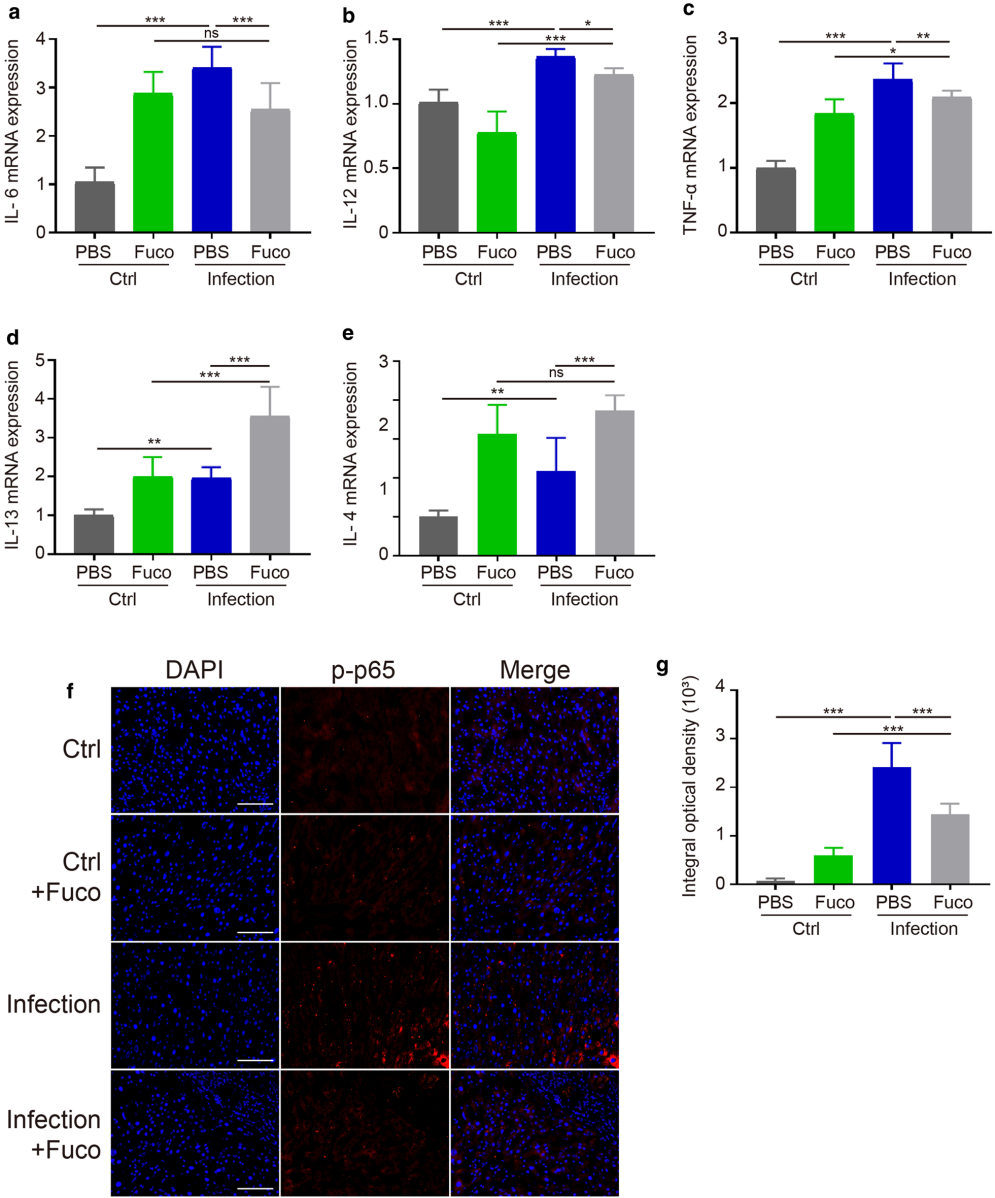
Fucoidan suppresses inflammation in the livers from *S. japonicum*-infected mice. Quantitative real-time PCR analysis of IL-6 (**a**), IL-12 (**b**), TNF-α (**c**), IL-13 (**d**) and IL-4 (**e**) in each group. The liver tissues of each group were prepared. The mRNA levels of inflammatory cytokines in the liver of each group were evaluated. The mRNA level of each gene was normalized to GAPDH mRNA levels in each sample. Results are presented using the 2^-ΔΔCq^ method. **f** Immunofluorescence of Pho-p65 in the liver tissue from normal or *S. japonicum*-infected mice treated with or without fucoidan. Original magnification: ×200. **g** The mean optical density of Pho-p65 positive cells from 6 random fields in each group was digitized and analyzed using Image-Pro Plus software. Data are expressed as the mean ± SD for each group (*n* = 5 for each group), and all experiments were performed twice with similar results. *P < 0.05, **P < 0.01, ***P < 0.001. *Abbreviation*: ns, not significant (ANOVA/LSD). *Scale-bars*: **f**, 50 μm

We next examined whether fucoidan treatment was associated with the infiltration of inflammatory cells in the livers from *S. japonicum*-infected mice. We found that the hepatic mRNA levels of IL-6, IL-12, TNF-α, IL-4 and IL-13 were significantly elevated in the *S. japonicum*-infected mice, relative to that in the control group (ANOVA test; IL-6: *F*_(3, 28)_ = 43.52, *P *< 0.0001; IL-12: *F*_(3, 23)_ = 45.33, *P *< 0.0001; TNF-α: *F*_(3, 24)_ = 69.21, *P *< 0.0001; IL-4: *F*_(3, 18)_ = 23.74, *P *< 0.0001; IL-13: *F*_(3, 28)_ = 34.52, *P *< 0.0001) (Fig. 2a–e). However, the production of pro-inflammatory cytokines (IL-6, IL-12 and TNF-α) in the liver tissues was significantly reduced in the fucoidan-treated *S. japonicum*-infected mice (Fig. 2a–c), while increased mRNA expression of anti-inflammatory cytokines (IL-4 and IL-13) was observed (Fig. 2d, e).

Given that NF-κB played a crucial role in the regulation of hepatic inflammation [20], we further investigated the expression of p-p65 (phospho-Ser536) in *S. japonicum*-infected mice with or without fucoidan treatment (Fig. 2f, g). The protein expression of hepatic p-p65 was significantly enhanced in the liver of *S. japonicum*-infected mice (ANOVA test; *F*_(3, 20)_ = 80.21, *P *< 0.0001). Meanwhile, treatment with fucoidan led to less p-p65 infiltration in the *S. japonicum*-infected mice, suggesting that fucoidan may play a key role in the inhibition of the NF-κB signaling pathway and inflammatory cytokine production. Altogether, these data demonstrate that fucoidan can drive cells biased towards anti-inflammatory immunophenotype in the liver *via* inhibiting the NF-κB signaling pathway during *S. japonicum* infection.

### Total CD3^+^CD4^+^ T cells are independent of fucoidan in *S. japonicum*-infected mice

**Fig. 3:**
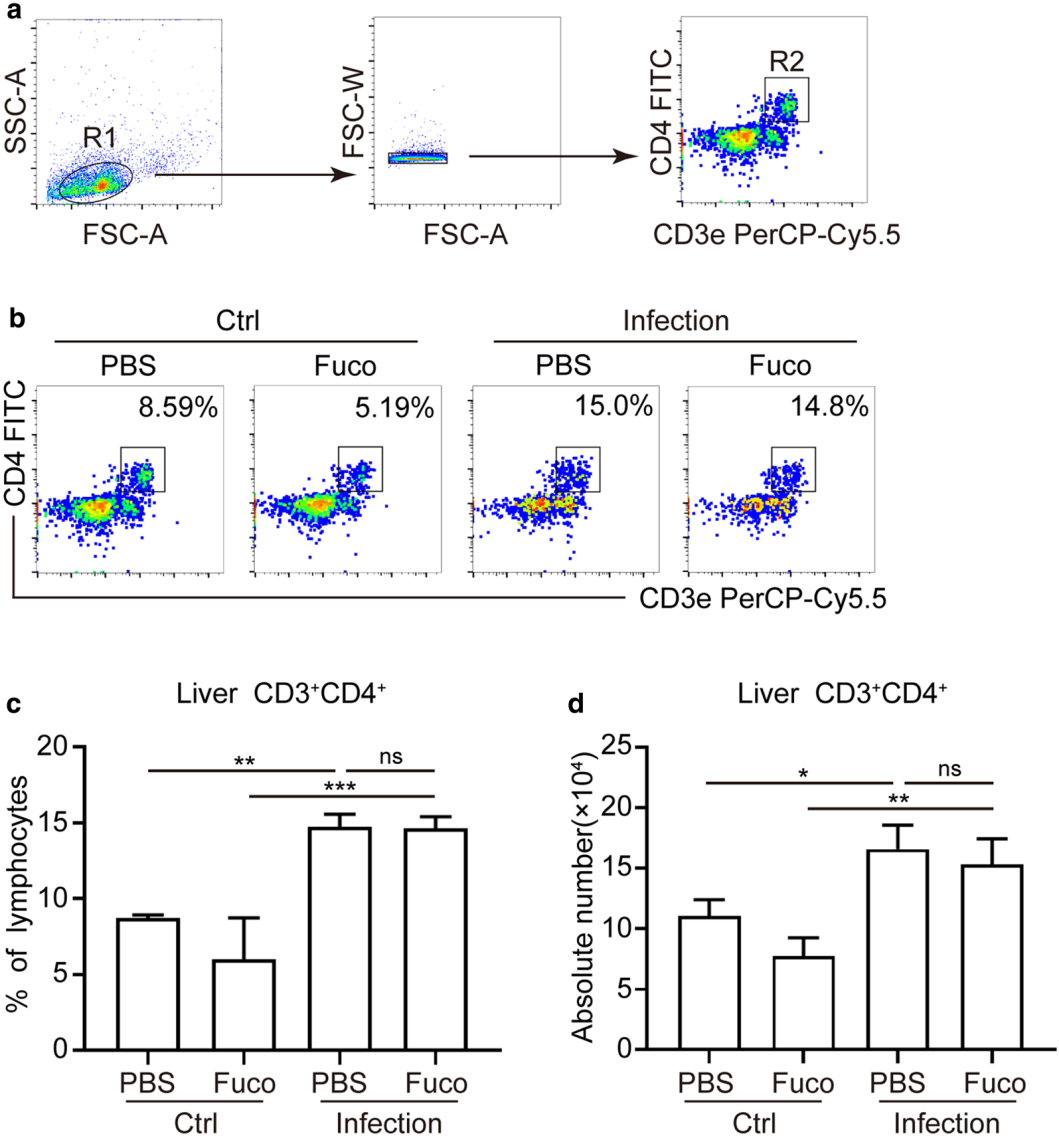
Flow cytometry analysis of total CD3^+^CD4^+^ T cells in mice. **a** Gating strategy for CD3^+^CD4^+^ T cells in the livers. **b** Representative flow cytometry gates from liver tissues are presented. **c**, **d** The percentage and the absolute number of CD3^+^CD4^+^ cells from hepatic lymphocytes of each group were analyzed by flow cytometry. Data are expressed as the mean ± SD for each group (*n* = 5 for each group), and all experiments were performed twice with similar results. *P < 0.05, **P < 0.01, ***P < 0.001. *Abbreviation*: ns, not significant (ANOVA/LSD)

As reported, adaptive CD4^+^ T cells are highly involved in immunopathology and the regulation of inflammation in schistosomiasis [21]. Thus, we next investigated whether total CD3^+^CD4^+^ T cells were regulated after fucoidan treatment during *S. japonicum* infection. As is shown in Fig. 3, Additional file 2: Figure S1, compared with the untreated control group, both percentage (ANOVA test; liver: *F*_(3, 8)_ = 26.00, *P* = 0.0002; spleen: *F*_(3, 13)_ = 35.32, *P *< 0.0001) and absolute number (ANOVA test; liver: *F*_(3, 8)_ = 18.81, *P* = 0.0006; spleen: *F*_(3, 13)_ = 18.81 *P* = 0.0001) of CD3^+^CD4^+^ T cells in livers and spleens were dramatically increased in the *S. japonicum*-infected mice. However, neither percentage nor the absolute number of CD4^+^ T cells changed after stimulated with fucoidan during *S. japonicum* infection in livers or spleens. These data corroborate that the effects of fucoidan attenuating hepatic pathology may have little association with the total CD3^+^CD4^+^ T cell proliferation in the liver and spleen from *S. japonicum*-infected mice.

### Fucoidan triggers Treg and Th2 cell responses in *S. japonicum*-infected mice

**Fig. 4:**
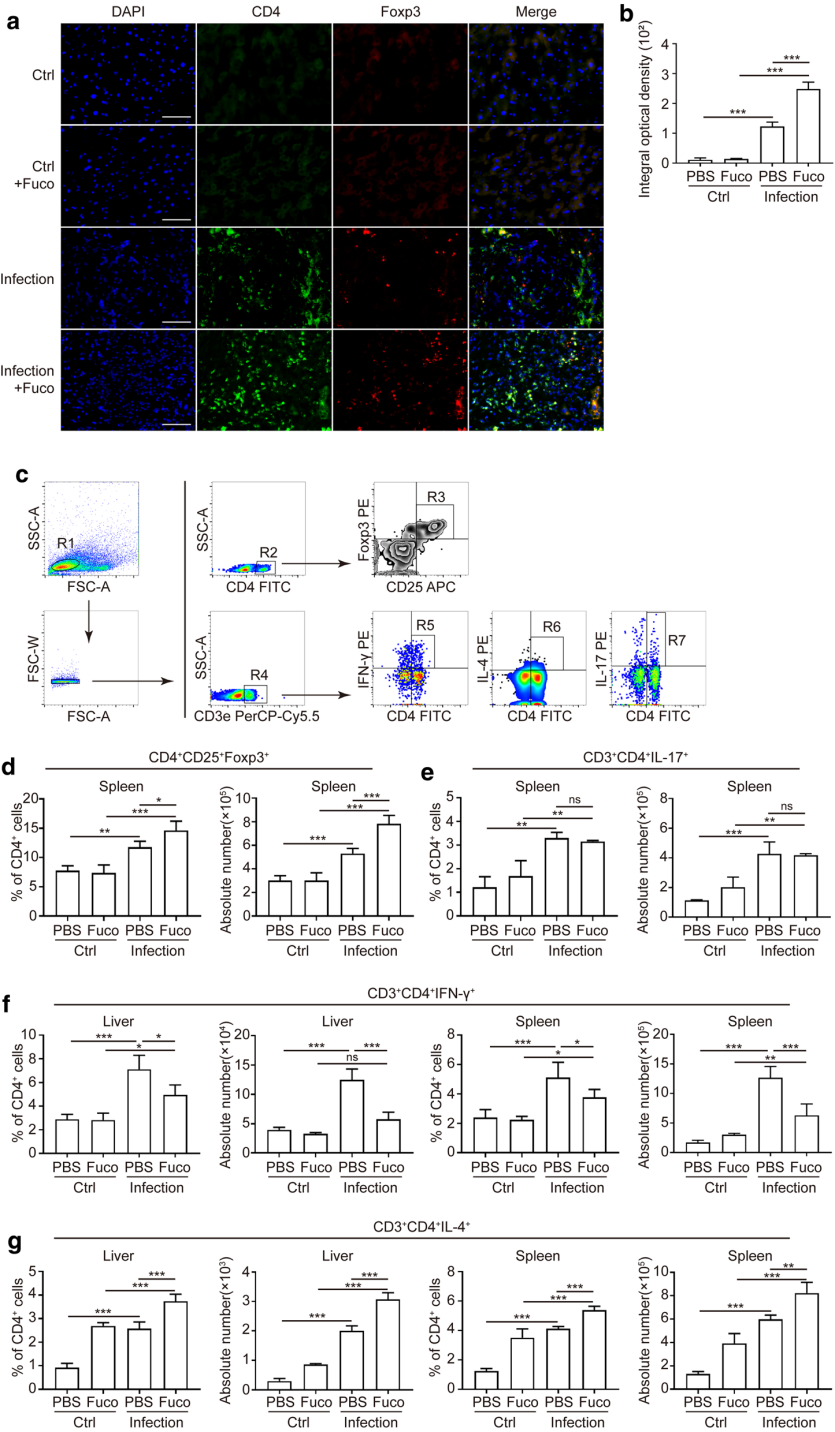
Analysis of CD4^+^ T cell subsets in mice after fucoidan treatment. **a** Immunofluorescence of CD4 (green) and Foxp3 (red) in the liver granulomas from the normal or *S. japonicum*-infected mice treated with or without fucoidan. Original magnification: ×400. **b** The mean optical density of CD4^+^Foxp3^+^ cells in each group was digitized and analyzed using Image-Pro Plus software. **c** Gating strategy for CD3^+^CD4^+^Foxp3^+^ (Treg), CD3^+^CD4^+^IL-17^+^ (Th17), CD3^+^CD4^+^IFN-γ^+^ (Th1) and CD3^+^CD4^+^IL-4^+^ (Th2) cells. Percentages and absolute numbers of Treg (**d**), Th17 (**e**), Th1(**f**) and Th2 **(g)** cells from hepatic or splenic lymphocytes of each group were analyzed by flow cytometry. Data are expressed as the mean ± SD for each group (*n* = 5 for each group), and all experiments were performed twice with similar results. *P < 0.05, **P < 0.01, ***P < 0.001. *Abbreviation*: ns, not significant (ANOVA/LSD). *Scale-bars*: **a**, 25 µm

We next illustrated whether treatment with fucoidan affects the CD4^+^ T cell subsets during *S. japonicum* infection. By using immunofluorescence (Fig. 4a), we found the upregulation of CD4^+^Foxp3^+^ Treg cells in the hepatic granulomas (ANOVA test; *F*_(3, 8)_ = 76.40, *P *< 0.0001) from fucoidan-treated *S. japonicum-*infected mice. Results also showed that Treg responses were significantly enhanced in spleens (ANOVA test; percentage in spleens: *F*_(3, 11)_ = 28.04, *P *< 0.0001; absolute number in spleens: *F*_(3, 11)_ = 57.30, *P *< 0.0001) (Fig. 4c, d), which was in accordance with the previous study that Treg cells played critical roles in regulating the development of immunopathology in schistosomiasis [18]. Moreover, there was also a trend for increased Th2 cells after fucoidan treatment (ANOVA test; percentage in livers: *F*_(3, 8)_ = 73.05, *P *< 0.0001; in spleens: *F*_(3, 11)_ = 90.83, *P *< 0.0001; absolute number in livers: *F*_(3, 8)_ = 195.9, *P *< 0.0001; in spleens: *F*_(3, 11)_ = 73.49, *P *< 0.0001) (Fig. 4c, g), whilst Th1 responses were considerably reduced (ANOVA test; percentage in livers: *F*_(3, 8)_ = 18.79, *P* = 0.0006; in spleens: *F*_(3, 11)_ = 15.63, *P *< 0.001; absolute number in livers: *F*_(3, 8)_ = 41.77, *P *< 0.0001; in spleens: *F*_(3, 11)_ = 45.48, *P *< 0.0001) (Fig. 4c, f). However, proportions or absolute numbers of Th17 cells in the spleens did not expand (Fig. 4c, e). Taken together, these phenomena suggest that fucoidan could play an imperative role in regulating CD4^+^ T cell subsets during *S. japonicum* infection, involving a mechanism of Treg and Th2 cell inducement.

### Fucoidan induces Treg and Th2 cell response *in vitro*

**Fig. 5:**
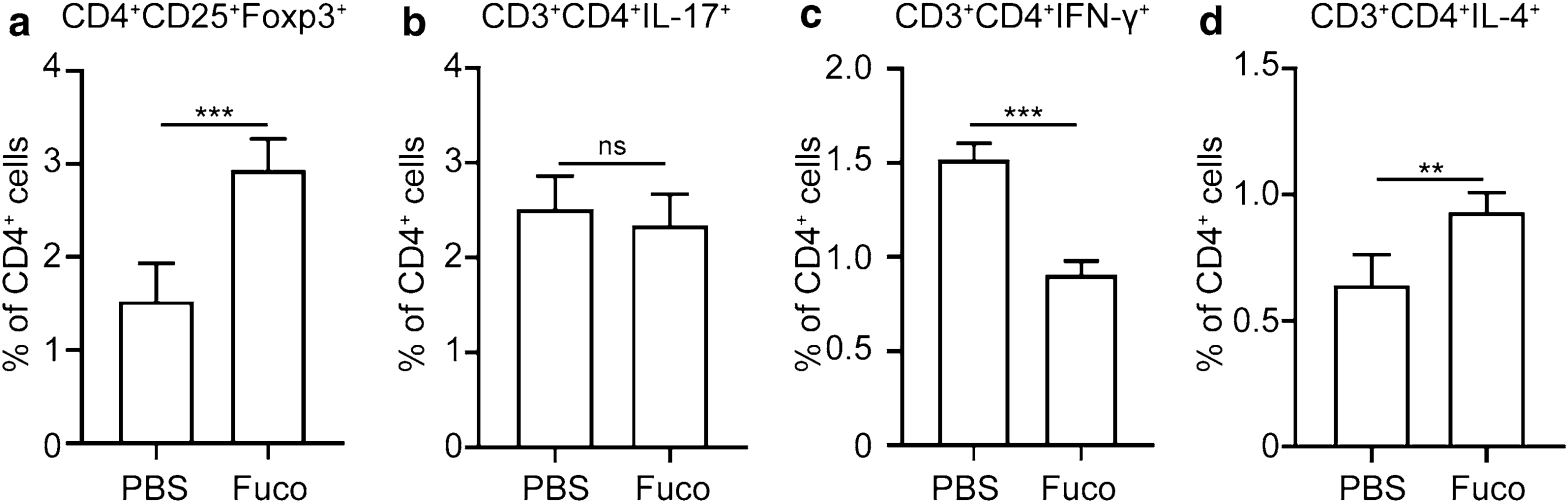
Flow cytometry analysis of subsets of CD4^+^ T cells *in vitro*. Percentage of Treg (**a**), Th17 (**b**), Th1 (**c**) and Th2 (**d**) cells were analyzed by flow cytometry. Data are expressed as the mean ± SD for each group (*n* = 5 for each group), and all experiments were performed twice with similar results. *P < 0.05, **P < 0.01, ***P < 0.001. *Abbreviation*: ns, not significant (Student’s t-test)

To further confirm the role of fucoidan in CD4^+^ T cell immune responses, splenocytes from normal WT mice were stimulated with fucoidan *in vitro* before flow cytometry analysis. Consistent with the *in vivo* data, *in vitro* treatment splenocytes with fucoidan led to an increase in Treg and Th2 cells and a decrease in Th1 cells (t-test; Treg: *t*_(8)_ = 5.983, *P* = 0.0003, Th2: *t*_(8)_ = 4.628, *P* = 0.002, Th1: *t*_(8)_ = 11.77, *P *< 0.0001) (Fig. 5a, c, d). Additionally, the proportion of Th17 cells was undifferentiated between PBS and fucoidan-treated groups (Fig. 5b).

### Fucoidan-induced immunosuppressive activity in Treg cells

**Fig. 6:**
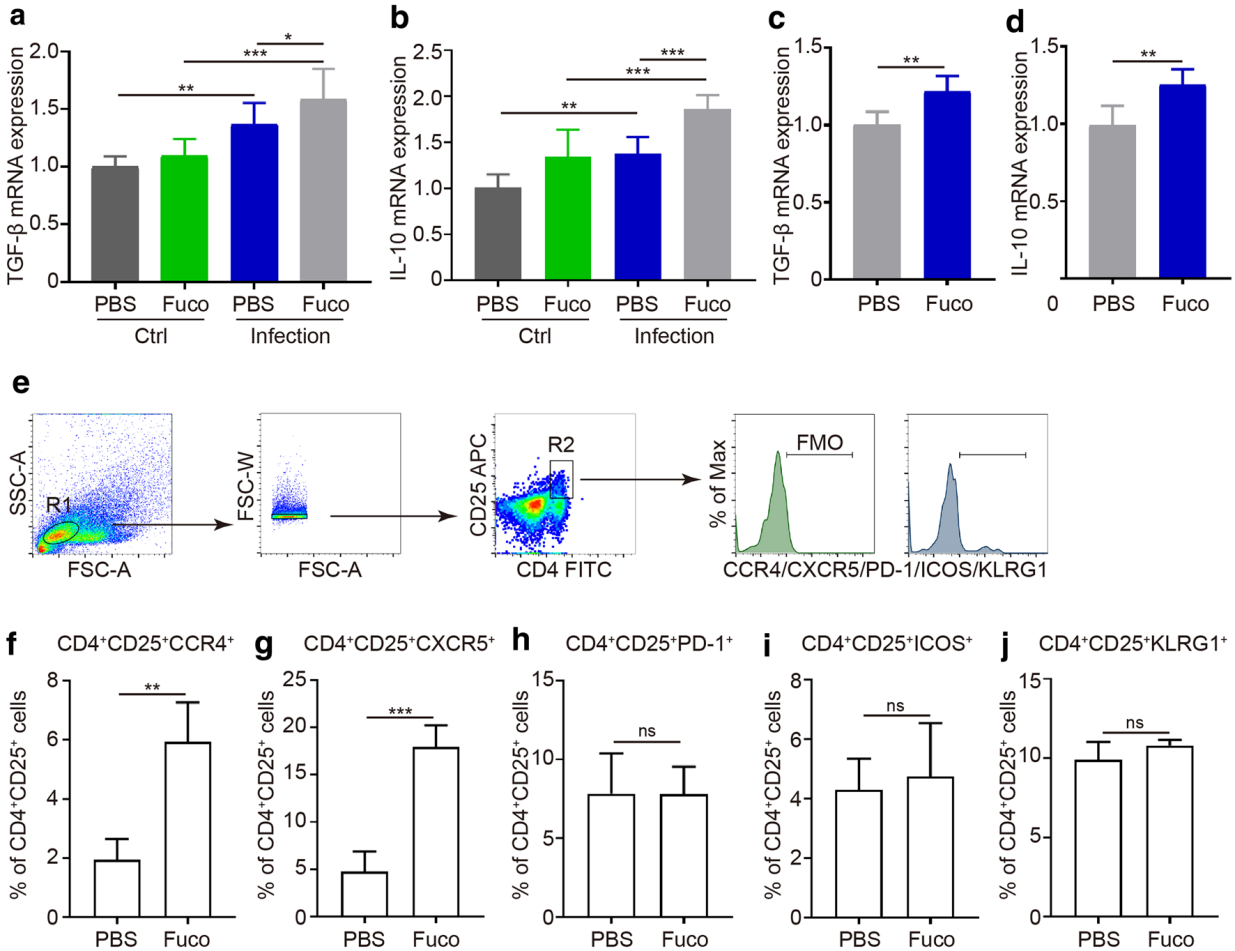
Fucoidan enhances immunosuppressive properties in Treg cells. qRT-PCR analysis of TGF-β and IL-10 in liver tissues (**a**, **b**) and RAW264.7 cells (**c**, **d**). The mRNA level of each gene was normalized to GAPDH mRNA levels in each sample. Results are presented using the 2^-ΔΔCq^ method. **e** Gating strategy for CCR4^+^, CXCR5^+^, PD-1^+^, ICOS^+^ and KLRG1^+^ Treg cells. Frequencies of CCR4^+^ (**f**), CXCR5^+^ (**g**), PD-1^+^ (**h**), ICOS^+^ (**i**) and KLRG1^+^ (**j**) Treg cells were analyzed by flow cytometry. All flow cytometry results were analyzed and plotted using Fluorescence Minus One controls (FMO). Data are expressed as the mean ± SD for each group (*n* = 5 for each group), and all experiments were performed twice with similar results. *P < 0.05, **P < 0.01, ***P < 0.001. *Abbreviation*: ns, not significant (Student’s t-test, ANOVA/LSD)

Treg cells have been widely acknowledged to suppress immune responses by secreting cytokines such as IL-10 and TGF-β [22]. In our study, we found that the hepatic mRNA levels of TGF-β and IL-10 were significantly elevated in the S. *japonicum-*infected mice when compared with normal WT mice, and a further increasing mRNA expression was observed in the fucoidan-treated S. *japonicum-*infected group (ANOVA test; TGF-β: *F*_(3, 23)_ = 15.72, *P *< 0.0001; IL-10: *F*_(3, 23)_ = 25.13, *P *< 0.0001) (Fig. 6a, b). Similar results were also observed *in vitro* stimulated macrophages, namely an uptrend after fucoidan treatment (t-test; TGF-β: *t*_(8)_ = 3.689, *P* = 0.006; IL-10: *t*_(8)_ = 3.617, *P* = 0.0063) (Fig. 6c, d).

A wide array of functional surface molecules on Treg cells assists Tregs in accomplishing their migratory, anti-inflammatory and immunosuppressive functions in the immune environment [23]. To investigate the suppressive function of Treg cells, we next determined surface molecules relatively specific to Treg cells induced by fucoidan (Fig. 6e). CCR4, the CC chemokine receptor principally involved in homing of T cells to inflammatory sites [24], increased on CD4^+^CD25^+^ Tregs in the presence of fucoidan compared to the control group (t-test; *t*_(6)_ = 5.326, *P* = 0.002) (Fig. 6f). CCR5 is also involved in immunosuppression and migration [25, 26], and significantly, the proportion of CCR5^+^ Tregs increased (t-test; *t*_(5)_ = 7.948, *P *< 0.001) (Fig. 6g). However, there were no remarkable differences in the other Treg-associated markers, such as PD-1, ICOS and KLRG1, after fucoidan treatment (t-test; PD-1: *t*_(6)_ = 0.02423, *P* = 0.98; ICOS: *t*_(6)_ = 0.4304, *P* = 0.68; KLRG1: *t*_(5)_ = 1.308, *P* = 0.25) (Fig. 6h-j). These results indicate that fucoidan has the potential immunosuppressive activity of Treg cells characterized by the expression of CCR4 and CXCR5.

### Fucoidan promotes anti-inflammatory response in macrophages

Since macrophages play a pivotal role in regulating pathogenesis in schistosomiasis japonica [2], we next investigated whether fucoidan could induce changes in macrophage response. Results showed that stimulation with fucoidan resulted in an M2-dominant phenotype, as evidenced by increased expression of anti-inflammatory cytokines (IL-4 and IL-13) (t-test; IL-4: *t*_(8)_ = 3.075, *P* = 0.02; IL-13: *t*_(7)_ = 3.210, *P* = 0.0149) (Additional file 3: Figure S2d, e), rather than classically activated macrophage (M1)-related cytokines (IL-6, IL-12 and TNF-α) (t-test; IL-6: *t*_(8)_ = 5.054, *P* = 0.0010; IL-12: *t*_(8)_ = 7.994, *P *< 0.0001; TNF-α: *t*_(8)_ = 3.852, *P* = 0.005) (Additional file 3: Figure S2a–c). These data suggest the potential role of fucoidan in mediating the anti-inflammatory effects during pathogen infection.

## Discussion

Fucoidan, mainly extracted from brown seaweed, is a complex sulfated polysaccharide that exhibits a wide spectrum of biological activities including anti-inflammatory, anticancer, antioxidant, anticoagulant, antiviral and immunomodulatory [27]. Especially in the modulation of immunity, fucoidan can be used as a tool to terminate various disease processes, such as viral and bacterial infections [28]. It has been reported that fucoidan can activate cytotoxic T cells, and further research demonstrates that fucoidan enhances dendritic cell maturation and promote immune response [29, 30]. These studies have highlighted fucoidan can attenuate the immunopathology by encouraging both innate and adaptive immune responses. However, to the best of our knowledge, how fucoidan regulates CD4^+^ T cells immune responses during *S*. *japonicum* infection has not been fully elucidated. We demonstrated for the first time that fucoidan could attenuate *S*. *japonicum*-induced liver pathology and blockage of the inflammatory response associated with the strong immunosuppressive function of Treg cells.

Schistosomiasis is a parasitic disease caused by infection with several species of schistosome trematodes, affecting over 200 million people worldwide [31]. During *S. japonicum* infection, parasite eggs are trapped within organs such as the liver, spleen and intestine, which contributes to severe granulomatous inflammation and fibrosis formation [3, 32]. An early report has demonstrated that fucoidan could attenuate CCl_4_-induced liver fibrosis [33]. However, to the best of our knowledge, there are no studies of fucoidan on liver disease resulting from natural infection. In the present study, granulomatous size and fibrosis response in the liver were significantly decreased after treated with fucoidan in a *S*. *japonicum*-infected mouse model. Therefore, we suppose that fucoidan plays a role in the suppression of hepatic immunopathology during natural pathogen infection.

The process of egg granuloma formation and fibrosis is dependent upon the immune microenvironment, particularly specific cytokine production [34, 35]. Here we observed an anti-inflammatory phenotype in the liver from the fucoidan-treated infection group, indicating that fucoidan could exert a suppressive influence on hepatic inflammation. The NF-κB signaling activation plays a pivotal role in inflammation induction and regulation [36, 37]. Activation of the NF-κB signal pathway had been reported to be closely associated with the development of hepatic granuloma and fibrosis [38]. Interestingly, fucoidan has been reported to attenuate the expression of NF-κB in the hepatic tissues from a NAFLD mice model [37]. Importantly, a recent study has also confirmed that fucoidan could inhibit LPS-induced inflammation and pro-inflammatory cytokine mRNA expression in macrophage by blocking the TLR4/NF-κB signal pathway [16]. Our study provided evidence that fucoidan may reduce liver granuloma and fibrosis at least partly due to the decreased TLR4/NF-κB signaling pathway, evidenced by the weakened expressed Pho-p65.

Numerous studies have shown that CD4^+^ T cells are the predominant contributors to inflammatory responses and the regulators of hepatic granuloma and fibrosis in schistosomiasis [18, 21]. Moreover, fucoidan has been reported to activate the growth of T and B cells in the spleen [39, 40], and we further speculated that fucoidan could alleviate hepatic immunopathology by affecting the subsets of CD4^+^ T cells. Remarkably, Treg cells are widely recognized to respond to local milieu to behave as subtypes that are equipped with distinct immunosuppressive programs to function in inflammatory settings. Corresponding with the previous study about Treg cells relieving hepatic inflammatory responses and immunopathology in schistosomiasis japonica [41], we found that fucoidan induced Treg cells expansion, which might contribute to the attenuated hepatic immunopathology during *S*. *japonicum* infection. Meanwhile, results of qRT-PCR also showed ascending expression of TGF-β and IL-10 both *in vivo* and *in vitro* treated with fucoidan, principally due to the enhanced immunosuppressive microenvironment induced by Treg cells. Previous studies showed that the process of granuloma depositing and fibrosis is triggered with an initial Th1-dominant response to a late Th2-dominant response [42, 43]. However, our data showed that Th2 cells were expanded, whereas Th1 cells were reduced in the presence of fucoidan during *S*. *japonicum* infection. We speculated that the abnormal Th1/Th2 response induced by fucoidan might play a subordinate role in this process.

To function properly, Treg cells regulate the activities of a wide range of cellular molecules, namely chemokine receptors, programmed cell death-1 (PD-1) and inducible co-stimulatory molecules (ICOS) [23]. The important role of these surface molecules and their association with inflammatory responses has been widely documented in both animal models and human samples. For example, CCR4-deficient Treg cells fail to migrate, causing severe inflammatory diseases in the lungs, intestines and other nonlymphoid organs [44, 45]. CXCR5^+^ follicular regulatory (Tfr) cells, an especially-classified thymic-derived Foxp3^+^ Tregs, are reported to increase within CD4^+^ T cells and total Tfh cells in schistosomiasis patients [46]. Tregs lacking CXCR5 show impaired suppressive function and cause enhanced germinal center responses [47]. In our findings, both CCR4^+^ and CXCR5^+^ Tregs increased after fucoidan treatment, while no variations were observed on the expressions of PD-1, ICOS, and KLRG1. Hence, the enhanced immunosuppressive activity induced by fucoidan may be related to the functional expressions of CCR4 and CXCR5 on Treg cells, thus relieving the hepatic inflammatory response and resultant process of granuloma and fibrosis.

The granulomatous immune response is also characterized by complicated cells, such as macrophages, which account for approximately 30% of granuloma cells [48, 49]. A previous study showed that IFN-γ, the product of Th1 cells and acting predominantly on macrophages to drive M1 differentiation, which secret various pro-inflammatory cytokines, such as TNF-α, IL-6 and IL-12, and activate the adaptive immune response [50]. High levels of IL-4 and IL-13, which can be secreted primarily by type-2 immune cells such as Th2 cells to act toward the alternative activation of macrophages into the M2 phenotype [50, 51]. Our *in vitro* study suggested that the increased M2 macrophages polarization induced by fucoidan was probably due to the enhanced Th2 cells, while the declined M1 macrophages related to the decreased Th1 cells. A previous study showed that fucoidan is the ligand of scavenger receptor A (SR-A) [52], which has been found to promote the liver pathology through inducing M2 macrophages and Th2 cell response during *S*. *japonicum* infection [10]. However, our data indicate the fucoidan attenuated liver pathology, one possible explanation is that fucoidan acts on not only SR-A but also TLR4 pathway [16], the latter of which activates the production of pro-inflammatory cytokines and might be the potential mechanism in the present study, which requires further elucidated in the future.

## Conclusions

In summary, this study indicates that fucoidan can effectively alleviate hepatic immunopathology during *S*. *japonicum* infection, one possible mechanism of which is that fucoidan regulates CD4^+^ T cells dominated by immunosuppressive functions of Tregs. Our data provide a novel insight into the biological functions of fucoidan from an immunomodulatory perspective, particularly its regulatory effects on the immune response.

## Supplementary information

**Additional file 1: Table S1.** The primer sequences used in detecting the levels of mRNA.

**Additional file 2: Figure S1.** Flow cytometry of total CD3^+^CD4^+^ T cells in spleens.

**Additional file 3: Figure S2.** The mRNA expression of cytokines in fucoidan-treated macrophages.

## Data Availability

The data that support the findings of this study are included in the article and its additional files.

## Abbreviations

CCR4: CC chemokine receptor type 4
CXCR5: CXC chemokine receptor type 5
IL: interleukin
NF-κB: nuclear factor-κB
TGF-β: transforming growth factor-β
Th cell: helper T cell
TNF-α: tumor necrosis factor-α
Treg cell: regulatory T cell
