# Supplementary material for: Therapeutic potential of fucoidan in the reduction of hepatic pathology in murine schistosomiasis japonica
Source: Parasit Vectors. 2020 Sep 7;13:451. doi: 10.1186/s13071-020-04332-7 (PMC7487607; doi:10.1186/s13071-020-04332-7)
Supplement: Supplementary file 1 — Additional file 1: Table S1. The primer sequences used in detecting the levels of mRNA. [file 13071_2020_4332_MOESM1_ESM.pdf]

Additional file 1: Table S1. The primer sequences used in detecting the levels of mRNA.

**Gene name**	**Gene ID**	**Forward (5'-3')**	**Reverse (5'-3')**
IL-4	16189	ATGGGTCTCACCTCCCAACTG	TCAGCTCGAACACTTTGAATAT
IL-6	16193	GAGGATACCACTCCCAACAGACC	AAGTGCATCATCGTTGTTCATACA
IL-10	16153	ACTTTAAGGGTTACTTGGGTTGC	ATTTTCACAGGGGAGAAATCG
IL-12p35	16159	GACAGTGGAGGCACCAGGCC	CAGACATCGCTGTCCCGGCG
IL-13	16163	GCCAGCCCACAGTTCTAC	GAGATGTTGGTCAGGGAAT
TGF-β	21803	ATGCTAAAGAGGTCAAAAGC	CCAAGGTAACGCCAGGAATT
TNF-α	21926	CATCTTCTCAAAATTCGAGTGACAA	TGGGAGTAGACAAGGTACAACCC
GAPDH	14433	GGTGAAGGTCGGTGTGAACG	ACCATGTAGTTGAGGTCAATGAAGG
