# Supplementary material for: Therapeutic potential of fucoidan in the reduction of hepatic pathology in murine schistosomiasis japonica
Source: Parasit Vectors. 2020 Sep 7;13:451. doi: 10.1186/s13071-020-04332-7 (PMC7487607; doi:10.1186/s13071-020-04332-7)
Supplement: Supplementary file 2 — Additional file 2: Figure S1. Flow cytometry of total CD3+CD4+ T cells in spleens. [file 13071_2020_4332_MOESM2_ESM.pdf]

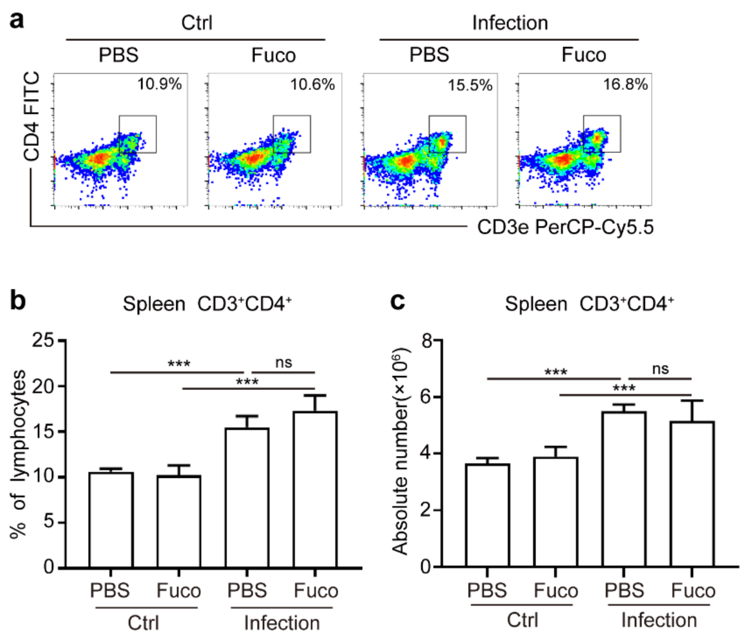

Additional file 2: Figure S1. Flow cytometry of total $CD3^{+}CD4^{+}$ T cells in spleens.

(**a**) Representative FACS gates from splenocytes are presented. (**b,c**) The percentage and the absolute number of $CD3^{+}CD4^{+}$ cells from splenocytes of each group were analyzed by flow cytometry. Data are expressed as the mean ± SD for each group, and all experiments were performed twice with similar results. **P*<0.05, ***P*<0.01, ****P*<0.001 (ANOVA/LSD).
