# Supplementary material for: Therapeutic potential of fucoidan in the reduction of hepatic pathology in murine schistosomiasis japonica
Source: Parasit Vectors. 2020 Sep 7;13:451. doi: 10.1186/s13071-020-04332-7 (PMC7487607; doi:10.1186/s13071-020-04332-7)
Supplement: Supplementary file 3 — Additional file 3: Figure S2. The mRNA expression of cytokines in fucoidan-treated macrophages. [file 13071_2020_4332_MOESM3_ESM.pdf]

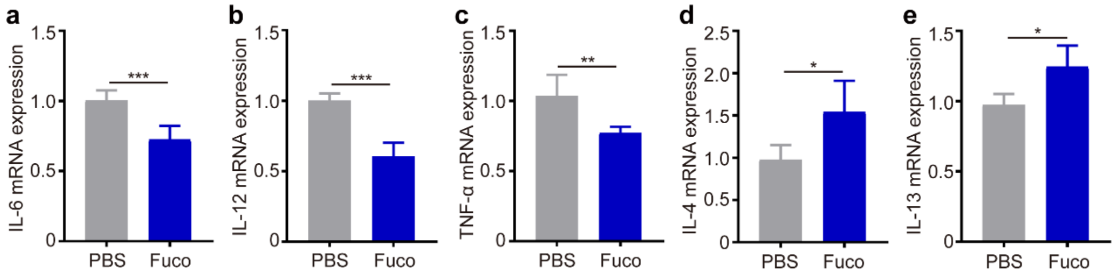

Additional file 3: Figure S2. The mRNA expression of cytokines in fucoidan-treated macrophages. The expression of inflammatory cytokines IL-6 (**a**), IL-12 (**b**), TNF-α (**c**), IL-4 (**d**) and IL-13 (**e**) were evaluated by RT-PCR. The expression level of each gene was standardized to the GAPDH mRNA levels in macrophages treated with PBS. Results are presented using the $2^{-\Delta\Delta Ct}$ method. Data are expressed as the mean ± SD for each group, and all experiments were performed twice with similar results. *P<0.05, **P<0.01, ***P<0.001 (Student's t-test).
